# Benign Prostatic Hyperplasia: A New Metabolic Disease of the Aging Male and Its Correlation with Sexual Dysfunctions

**DOI:** 10.1155/2014/329456

**Published:** 2014-02-13

**Authors:** Giovanni Corona, Linda Vignozzi, Giulia Rastrelli, Francesco Lotti, Sarah Cipriani, Mario Maggi

**Affiliations:** ^1^Endocrinology Unit, Medical Department, Azienda Usl, Maggiore-Bellaria Hospital, Bologna, Italy; ^2^Sexual Medicine Andrology Unit, Department of Experimental, Clinical and Biomedical Sciences, University of Florence, Viale Pieraccini 6, 50139 Florence, Italy

## Abstract

Metabolic syndrome (MetS) is a well-recognized cluster of cardiovascular (CV) risk factors including obesity, hypertension, dyslipidemia, and hyperglycaemia, closely associated with an increased risk of forthcoming cardiovascular disease and type 2 diabetes mellitus. Emerging evidence indicates that benign prostate hyperplasia (BPH) and its related lower urinary tract symptoms (LUTS) represent other clinical conditions frequently observed in subjects with MetS. Several modifiable factors involved in MetS determinism, such as inadequate diet, lack of physical exercise, and smoking and drinking behaviours are emerging as main contributors to the development of BPH. The pathogenetic mechanisms underlying the connection between MetS and BPH have not been completely clarified. MetS and its components, hypogonadism, and prostate inflammation probably play an important role in inducing BPH/LUTS. Although historically considered as a “normal” consequence of the aging process, BPH/LUTS should now be faced proactively, as a preventable disorder of the elderly. Type of diet and level of physical activity are now considered important factors affecting prostate health in the aging male. However, whether physical exercise, weight loss, and modifications of dietary habit can really alter the natural history of BPH/LUTS remains to be determined. Further research is advisable to better clarify these points.

## 1. Introduction

Time is an absolute dimension which ranks events as past, present, and future. Since biological aging is the accumulation and stratification of time-associated changes in a person, aging is an inevitable phenomenon, and, as such, it must be accepted. Because rejuvenation is impossible, the healthcare intervention in aging should be focused on formatting this biological process as an acceptable lifetime season and, therefore, as healthy as possible. We strongly believe that acting on modifiable factors—such as going to the primary care doctor routinely, a healthy diet, or smoking cessation—can reduce an individual's absolute propensity to aging. In contrast, chronic morbidities—such as cardiovascular diseases (CVD), type 2 diabetes mellitus (T2DM), osteoarthritis, and mental disabilities—are conditions that seniors often face as they age and that impair their enjoyment of this late lifetime season. Low-grade inflammation is supposed to represent the common determinant underlying almost all the aforementioned, age-related, and degenerative health conditions [1]. In fact, almost 10 years ago, Time magazine, on its cover, labeled inflammation as “The Secret Killer” for human health (http://content.time.com/time/magazine/article/0,9171,993419,00.html). However, inflammation per se is a beneficial reaction of the body, and its innate immune system, to an injurious stimulus, recognized 2000 years ago in the pioneering work of Celsius.

The concept of metabolic syndrome (MetS) was introduced almost 60 years ago, but only recently it was recognized as a valid construct to cluster some common medical disorders—such as visceral obesity, glucose intolerance, hypertension, and dyslipidemia—which increase the odds for CVD and T2DM [2]. Even in the case of MetS, chronic, low-grade inflammation is considered, in concert with insulin resistance, the milestone of the syndrome. In the male, three other bothersome, age-related conditions were recently proposed as new factors often associated with MetS [2–4]. They are hypogonadism, erectile dysfunction (ED), and benign prostate hyperplasia (BPH). These age-associated medical conditions have a relatively high socioeconomic burden and are generally not regarded as preventable ailments. In contrast, we strongly believe that their impact on male aging can be halted by lifestyle changes or at least buffered by available medications. In this study we will overview pathogenetic interconnections between BPH, inflammation, MetS and hypogonadism, highlighting possible interventions to prevent their negative effect on men's health. In fact, several modifiable factors, such as inadequate diet, lack of physical exercise, and smoking and drinking behaviors, are emerging as main contributors to the development of MetS and its related disorders, including BPH.

## 2. BPH/LUTS and Hypogonadism

Androgens play an essential role in the development and growth of the entire male genital tract and in particular of the prostate, stimulating *differentiation* and* proliferation* of both the epithelial and the stromal compartments of the gland. Androgens acts through activation of androgen receptor (AR), which is expressed in both prostatic stromal and epithelial cells.

### 2.1. Androgens and Prostate Differentiation

The differentiating and growth-promoting actions of androgens are exerted starting in early embryonic life and still persist in adulthood and senescence. In fetal life, the AR-induced differentiation and branching morphogenesis was deeply explored by the Cunha laboratory, which demonstrated the role of androgens in mesenchyme cell-induced prostatic development [5, 6]. Cunha et al. [7, 8] found that androgens could stimulate prostatic epithelial development and growth interacting with AR within the stromal tissue, under the influence of specific growth factors. This concept was originally based on tissue recombinant experiments, composed of wild-type stromal cells and AR-deficient epithelium from the testicular feminization mouse. During prostatic development, several growth factors, termed andromedins (IGF-1, EGF, and several FGF-related proteins), have been proposed to be the paracrine mediators of these androgen-induced, stromal-mediated, generation of prostatic epithelial buds, and subsequent ductal elongation and branching morphogenesis [9]. In the adult prostate, AR expression drives basal epithelial cells of the glands into differentiation to generate intermediate cells and into terminal differentiated luminal cells [10]. As a caveat of these prodifferentiating actions of androgens, recent studies indicate that hypogonadism is associated with a more aggressive phenotype of prostate cancer [11].

### 2.2. Androgens and Prostate Growth

Besides differentiation, another biological action of androgens in the prostate is to promote growth [12], which is essentially orchestrated in three distinct waves. The first growth wave is completed at birth, when the average weight of the prostate is about 1.5 grams. Prostatic development at this stage is a clear function of androgen signaling and is dependent on the function of the fetal testis. After a quiescent phase, at puberty—under the influence of increasing testosterone—the second wave starts: the prostate size reaches approximately 10 grams at early puberty and almost double that around the age of twenty [13]. Thereafter, the size of the prostate remains constant until midlate adulthood. At that time, in contrast to the pubertal growth phase which involves the entire gland, often there is a third selective growth phase, involving one of the three anatomically distinct prostate zones, the periurethral one, and which gives rise to BPH. BPH is a condition extremely prevalent in male adulthood and senescence, affecting 42% of men in the fifth decade to almost 90% in men older than 80 years [14]. BPH is essentially a histological diagnosis, characterized by hyperproliferation of the stromal and, to a lesser extent, of the epithelial compartment of the prostate, which can be clinically manifested as benign prostate enlargement (BPE), in almost half of the cases, or, less often, as benign prostate obstruction (BPO). The latter two clinical entities are characterized by progressive development of symptoms (lower urinary tract symptoms, LUTS), that are derived from prostate enlargement to the point where urination becomes difficult (BPE) or impaired (BPO), due to mechanical pressure on the bladder and urethra. Approximately 25% of men in their 50s and 80% of men in their 70s have clinically significant LUTS. However, not all men with BPH develop LUTS. In addition, not all men with LUTS have BPH as the underlying cause, because they are not disease-specific, being often multifactorial.

Although an increased androgen signaling is clearly implicated in the first two waves of prostate growth, its role in the third phase, BPH, is a matter of debate. In fact, a clear dose-response relationship between circulating androgen levels and BPH has never been demonstrated [15, 16]. In addition, during male senescence, androgens tend to decrease and not to increase [17]. Several recent studies indicate that a low testosterone (T), more than a high T, might have a detrimental effect on prostate biology. In fact, LUTS can even be lessened by androgen supplementation in hypogonadal men [18–25]. Recent data indicate that not only low testosterone but also high estradiol can favor BPH/LUTS progression. It is important to note that circulating T is actively metabolized to estrogens and part of T hormonal activity depends upon its binding to the estrogen receptors (ERs), that are present in both the prostate and bladder [26]. In addition, the enzyme P450 aromatase which converts androgens to estrogens [27] is highly expressed not only in fat tissue but also in the urogenital tract [28]. Evidence of an increased estrogen/androgen ratio was originally provided by Marmorston et al. almost half a century ago [29] reporting that the estrogen/androgen ratio in 24-hour urinary collections was elevated in men with BPH, as compared to normal controls. Several studies have observed a correlation between plasma 17*β*estradiol (17*β*E_2_) levels and prostate volume or other features of BPH [30–32], while others have not [33]. In two longitudinal, population-based cohort studies it was recently shown that a higher baseline 17*β*E_2_ was associated with a worse forthcoming maximal flow rate and urinary symptoms [34, 35].

## 3. BPH/LUTS and Metabolic Syndrome

The historical view that BPH-related LUTS are merely generated by the compression of the urethra through the volumetric increased transitional prostate has been heavily challenged in the last few years [36]. In fact, nowadays, BPH/LUTS are not only viewed as a mere hydraulic problem, to be solved by a surgical intervention, but as a metabolic problem, to be solved with a multidisciplinary approach, which includes also the endocrinologist. Several recent studies have provided convincing evidence of a possible role of MetS, and/or its individual components, in the development of BPH, prostate growth, and worsening of LUTS [36].

### 3.1. Hyperinsulinemia, Glucose Intolerance/T2DM, and BPH

**Figure 1:**
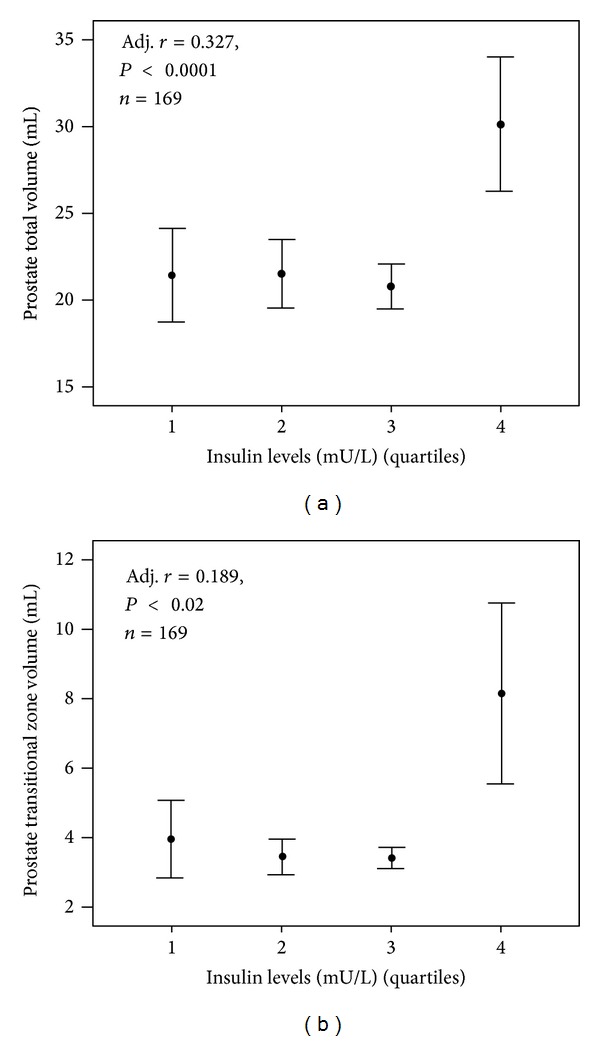
Association between insulin levels and prostate total or transitional zone volume. Association between insulin levels and prostate total (a) or transitional zone (b) volume. Insulin levels are reported as quartiles. All data are adjusted for age and total testosterone. Data are derived from a series of subjects seeking medical care for coupler infertility at our unit. The number of subjects with available parameters is reported in the inset. Note that the statistical analyses have been performed using insulin levels as a continuous variable, even if grouped in quartiles for graphical purposes.

Possible links between BPH and T2DM were noted, in a retrospective study, as far back as 1966 [37]. Since that time, hyperinsulinaemia/glucose intolerance (the key component of MetS) and even T2DM have been considered as potential risk factors for BPH/LUTS based on several studies [38, 39]. In a population-based cohort of African-American men aged 40–79 years, BPH patients reporting a diabetes history have a 2-fold increase in the risk of moderate-severe LUTS [40]. In diabetic individuals, a similar odds ratio for having LUTS was reported in the second Nord-Trondelag Health Study [41]. In a stepwise regression analysis, Nandeesha et al. [42] found that insulin levels were an independent predictor of prostate volume in symptomatic BPH patients aged over sixty. Interestingly, a similar conclusion was drawn by us in a sample of 171 young subjects undergoing transrectal sonography for couple infertility and not complaining of LUTS [43]. We found an association between prostate volume and insulin levels, which was retained after adjusting for total testosterone, other metabolic factors, and blood pressure [43]. All these findings indicate that insulin is an independent risk factor for BPH, most probably stimulating prostate growth acting on IGF receptors [44].  Figure 1 shows the relationship between increasing insulin levels (represented as quartiles) and prostate total and transitional zone volume, as detected by transrectal sonography, in the sample of subjects consulting for couple infertility, collected as previously described [43]. The highest quartile of insulin levels is associated with a clear increase in prostate size.

### 3.2. Obesity and BPH

In worldwide conducted studies, obesity—and in particular visceral obesity—was found to be often comorbid with BPH [45–49]. Although there were also some negative reports [50, 51], a recent meta-analysis, including a total of 19 studies, reported a positive association between BMI and LUTS associated with BPH (odds ratio (OR) = 1.28%) [52]. In population-based case-control studies, a marginal positive association was observed between risk of BPH and increased BMI. [52]. The impact of obesity on prostate size is apparent even in early adulthood, as demonstrated by a sonographic study conducted in 222 young men seeking medical care for couple infertility [53].

### 3.3. Dyslipidemia and BPH

The prostate synthesizes cholesterol at a level similar to the liver and accumulates it in a deposit within the gland in an age-dependent manner [54]. More than 70 years ago, Swyer [55] analyzed the cholesterol content in the prostate of BPH subjects and reported that its concentration was twice that in a normal prostate. Later on, Nandeesha et al. [56] reported that circulating total and HDL cholesterol were associated, in a positive and negative manner, respectively, with prostate enlargement in a series of 50 symptomatic BPH cases and 38 controls. However, other studies did not confirm the association [57–59]. In the Rancho Bernardo cohort study, Parsons et al. [60] found a 4-fold increased risk of BPH among diabetic men with elevated low density lipoprotein (LDL) cholesterol, but not in the overall cohort. This observation suggests that dyslipidemia per se is not sufficient enough to concur with BPH determinism, but the presence of other metabolic derangements, like T2DM, favors the process, because of an unfavorable total and LDL cholesterol particle size and density [60].

### 3.4. Hypertension and BPH

An association between BPH, hypertension, and T2DM was originally reported in a retrospective study 50 years ago [61]. Later on, hypertension was associated with increased odds of surgery for BPH in the Physician's Health Study [62] and with a higher prevalence of LUTS in other studies [63–65]. However, because both hypertension and BPH prevalence increase as a function of aging, the relationship between the two conditions was underlooked.

### 3.5. Metabolic Syndrome and BPH

From all the previous considerations (see Sections 3.1–3.4) it can be derived that each individual factor of MetS has been associated in some study with BPH/LUTS prevalence or progression, although several authors noted that their clustering, more than their individual presence, underlies the link. In 1998, Hammarsten et al. [66] elaborated this concept investigating the relationship between prostate volume and individual MetS components in 158 men with BPH, demonstrating that T2DM, hypertension, obesity, high insulin, and low HDL-cholesterol levels were all risk factors for the development of BPH. Thereafter, only few additional studies, based on the concept of the MetS construct, have investigated the association between MetS and BPH/LUTS; results are summarized in Table 1 [4, 67–73]. All the studies found an association between the presence of MetS, even if defined with different criteria, and prostate volume, whereas the relationship between MetS and LUTS is more controversial (see Table 1). However, changing definition of MetS has little impact on its long-term metabolic and CV consequences [74, 75]. In the study of Gacci et al. [4], reduced HDL-cholesterol and increased triglyceride levels were noted to be the main determinants of MetS-related prostate alterations.

A recently published epidemiological survey of the Boston area (BACH) confirmed an association between MetS and LUTS; however, when subjects were stratified by age, the association was confirmed only in the youngest individuals [76].

In the previously mentioned cohort of relatively young male subjects examined for couple infertility we recently reported a stepwise, component-dependent association between increasing MetS severity and prostate enlargement at color Doppler ultrasound (CDU) [43]. No association between MetS-related prostate CDU abnormalities and semen parameters was detected, even though, in this cohort, MetS was associated with poor sperm morphology [43, 77]. Increased central obesity and reduced HDL cholesterol were the main correlates of prostate enlargement in this young, asymptomatic population. This and previous evidence suggest that, beginning at a young age, MetS and in particular high waist circumference and reduced HDL cholesterol play an important role in prostate overgrowth. Interestingly, no association between MetS severity and prostate-related symptoms was observed, using either IPSS or the National Institutes of Health Chronic Prostatitis Symptom Index (NIH-CPSI) [43], which is a brief self-reported questionnaire for screening prostatitis symptoms [78].

## 4. BPH/LUTS and Inflammation

### 4.1. Epidemiological Evidence

In the last decade, cross-sectional and longitudinal observation of several large cohorts have finally confirmed that chronic inflammation is a crucial component of BPH pathogenesis. An examination of baseline prostate biopsies in a subgroup of 1,197 patients, followed for more than 4 years in the Medical Therapies of Prostate Symptoms (MTOPS) study to assess BPH-disease progression, found that men in the placebo arm with inflammation were significantly more likely to experience BPH worsening and at higher risk of acute urinary retention (AUR) or BPH-related surgery than those without [79]. This was confirmed in the subgroup analysis of 8,224 men in the Reduction by Dutasteride of Prostate Cancer Events (REDUCE) trial indicating that histologic inflammation was present in more than 78% men and that the severity of LUTS and the intensity of inflammation were related [80]. Another study retrospectively reviewed all histopathological examinations of 3,942 patients with BPH and showed that 43% of patients had histologic inflammation and 69% of them had chronic inflammation. In addition, inflammation in the prostate increased significantly with the increase in prostate volume and age [81]. Finally, the data from the placebo arm (1359 men) of the Prostate Cancer Prevention Trial (PCPT) demonstrated that circulating levels of inflammatory markers, including elevated CRP and interleukin-6 (IL-6), were associated with risk of incident, symptomatic BPH [82].

### 4.2. Physiopathology

Within the prostate, several classes of immunocompetent cells (lymphocytes, macrophages, and granulocytes) are physiologically resident and termed human prostate-associated lymphoid tissue (PALT). Activation of the intraglandular immune system PALT is the usual response to infectious agents. However, we believe that this initial acute inflammation could be succeeded by chronic inflammation that persisted if favored by hormonal and metabolic derangements or by exposure to other environmental and dietary factors [83]. Activated PALT recruits and stimulates the proliferation of other immunocompetent cells leading to an upregulation of several proinflammatory chemokines and cytokines [84]. Prostatic stromal cells—acting as targets of bacterial or viral toll-like receptor (TLR) agonists and, later on, as antigen-presenting cells (APC)—play a crucial role in the induction of inflammatory responses. They in fact activate CD4+ lymphocytes and favor their differentiation to the effector subsets Th1 and Th17 [85]. In addition, TLR activation leads to the production of proinflammatory cytokines (IL-6) and chemokines (IL-8 and CXCL10) capable of recruiting CXCR1- and CXCR2-positive leukocytes and CD15+ neutrophils and further promoting prostate cell hyperplasia, through the direct action of IL-8 or the release of other intraprostatic growth factors, like basic FGF [85–87]. Stromal BPH cells are able to secrete IL-8, CXCL-10, and IL-6 not only in response to specific proinflammatory stimuli (i.e., TNF*α* or the TLR 4 agonist lipopolysaccharide), but also to metabolic insults and, in particular, to oxidized LDL and insulin. This suggests the hypothesis that lipids can induce and sustain an inflammatory response in human prostatic cells [87, 88].

### 4.3. Clinical Evidence

In line with this preclinical evidence, in a multicentre study on 271 consecutive men treated with simple prostatectomy, we demonstrated that the presence of MetS—and in particular of some of its components, such as dyslipidemia—is associated with a more severe intraprostatic inflammation [87, 88]. In particular, histopathological examination of BPH specimens demonstrated that the inflammatory score (IS), as well as the positivity for the pan leukocyte marker CD45, significantly increased as a function of MetS components [86–88]. Among MetS components, reduced HDL cholesterol and elevated triglycerides were significantly associated with elevated IS and CD45 positivity. Fats could have, therefore, a detrimental effect on prostate cells, boosting prostate inflammation, a key factor in the development and progression of BPH/LUTS. In the previously mentioned cohort of young, infertile subjects [43], we noted a significant, stepwise correlation between the number of MetS components and seminal IL-8, which has been proposed as a surrogate marker of prostate inflammation [89–92]. In addition, in the same population, a higher MetS severity was associated with sonographic features of prostate inflammation, including texture nonhomogeneity, major calcification size, and elevated arterial peak systolic velocity. Abdominal adiposity and dyslipidemia were the main determinants, among MetS factors, of sonographic alterations and increased seminal IL-8 [43].

### 4.4. Experimental Models of MetS and Prostate Inflammation

**Figure 2:**
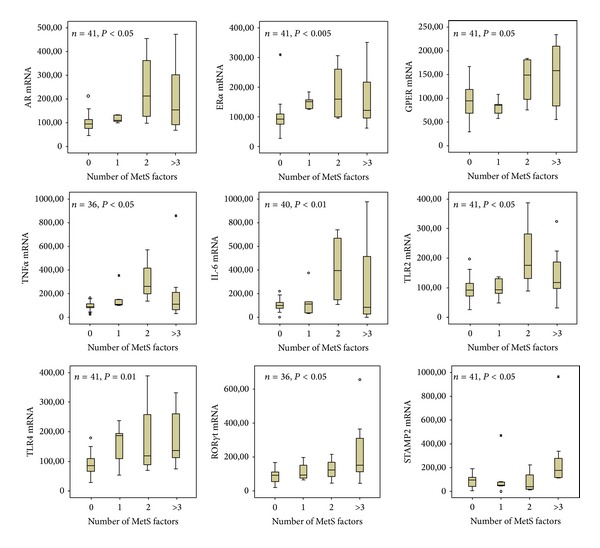
Gene expression of sex steroid receptors (upper panel) and inflammatory markers (middle and lower panels) in prostate of rabbits fed a regular diet (RD) or a high fat diet (HFD), according to metabolic syndrome (MetS severity). MetS severity was categorized as previously described [138], according to the number of factors present (abscissa). Ordinate axis indicates level of mRNA expression in arbitrary unit, as derived from quantitative RT-PCR analysis of the indicated prostate samples. Level of significance was derived from Kruskall-Wallis analysis of the data.

We recently developed an animal model of MetS by feeding New Zealand male rabbits a high fat diet (HFD) for 12 weeks. MetS in rabbits was characterized by glucose intolerance, dyslipidemia, hypertension, increased visceral fat accumulation, and hypogonadotropic hypogonadism with a concomitant hyperestrogenism [93–95]. In MetS animals we have described a specific prostate [96] and bladder [93] phenotype, which includes features of inflammation, tissue remodeling, and hypoxia. Interestingly, almost all these alterations were positively associated with a low-testosterone and high-estrogen milieu [93, 96, 97]. Accordingly, Figure 2 (upper panel) shows that MetS severity, in rabbit fed HFD or a regular diet (RD), is associated with a stepwise increase in AR and ER*α*, but not ER*β* (not shown), gene expression within the prostate. In addition, in the same figure, it is shown that also the nonclassical, G protein-coupled estrogen receptor, GPER/GPR30, increases as a function of number of MetS factors. This indicates a potentially increased sensitivity of the MetS prostate to changing sex steroids. We, in fact, found that T administration to HFD rabbits reverted the majority of MetS-induced prostate alterations [96]. This finding is in line with the observation that, in human BPH stromal cells, the selective AR agonist DHT was able to blunt TNF*α*, LPS, or CD4(+)T cell-induced secretion of inflammatory/growth factors, including IL-6, IL-8, and bFGF, by blocking NF-*κ*B nuclear translocation [86]. A protective effect of DHT was also found on oxLDL- or insulin-induced IL-8 secretion [87]. Interestingly, DHT was also able to prevent TNF*α*-induced LOX-1 (the receptor for oxLDL) mRNA expression. This strongly indicates a potential beneficial effect of AR signaling on diet-induced prostate inflammation. In contrast, tamoxifen dosing to HFD rabbits further exacerbated MetS-induced prostate alterations, most probably by stimulating GPER/GPR30, as demonstrated by experiments with selective ligands for these receptors and by genetic ablation of their expression [26].

We also recently reported that the prostate of HFD-rabbits showed an increased expression of both mRNA and protein for phosphodiesterase type 5 (PDE5), the enzyme that catalyzed cGMP breakdown [97]. PDE5 expression within the prostate was associated with the majority of HFD-induced markers of inflammation, fibrosis, and myofibroblast activation, and, in particular, with COX2 and TNF*α* among inflammatory genes and with TGF*β*, ROCK2, and *α*SMA among those genes specifically involved in fibrosis and myofibroblast activation. Interestingly, HFD-induced PDE5 overexpression was counteracted by T dosing. Consistent with this effect, a negative correlation between prostate PDE5 mRNA expression and plasma testosterone/estradiol ratio was identified. However, a direct role of hypogonadism in HFD-induced PDE5 upregulation was ruled out by the observation that hypogonadotropic hypogonadal rabbits (GnRH analog-treated group), characterized by a reduced plasma testosterone/estradiol ratio, showed prostatic PDE5 mRNA expression similar to that of the RD group, which was not modified by T treatment [97]. Hence, we can hypothesize that HFD-related derangements, rather than hypogonadism per se, may be related to the PDE5 overexpression in the prostate.

## 5. Possible Intervention in MetS-Associated BPH/LUTS

Current therapy for BPH/LUTS is largely based on the use of *α*_1_-adrenergic receptor blockers, which relax prostatic smooth muscle, and 5-*α* reductase inhibitors, which reduce prostatic volume. Accordingly, current EAU guidelines attributed level of evidence of 1b and 1a to *α*_1_-blockers and 5-*α* reductase inhibitors, respectively, for the treatment of men with moderate-to-severe LUTS [98]. Recently, the possible use of PDE5 inhibitors was also recognized as a valuable treatment of the condition, with a level of evidence of 1b [98]. However, the same guidelines also suggest the usefulness of lifestyle modifications, without better explanation except for avoidance or moderation of caffeine or alcohol intake that may have a diuretic and irritant effect, thereby increasing fluid output and enhancing frequency, urgency, and nocturia [98].

### 5.1. Lifestyle Modification

Current evidence, suggesting a close relationship among BPH/LUTS, MetS, hypogonadism, and inflammation, indicates that the impact of lifestyle modification should be more carefully analyzed. Prospective data of the Health Professionals Follow-up Study (HPFS), on more than 18,000 men without LUTS at baseline, recently showed that men with higher total and abdominal adiposity or who gained weight at follow-up were more likely to develop LUTS or experience progressive LUTS [99]. Previous meta-analyses have clearly demonstrated that lifestyle modifications, such as weight loss and increased consumption of fruit and vegetables, can reduce the incidence of obesity-related morbidities including hypogonadism [100], type 2 diabetes [101], coronary artery disease [102], and stroke [103]. Quite unexpectedly, studies on efficacy of lifestyle modifications on BPH/LUTS outcome are still lacking.

In 2002, Suzuki et al. [104] first reported that men with high energy intakes and particularly with high consumption of protein and polyunsaturated fatty acid were at a greater risk of developing BPH. Data from the placebo arm in the Prostate Cancer Prevention Trial (PCPT), which enrolled 18,880 men aged over 50 years, confirmed that high consumption of red meat and a high fat diet increased the risk of BPH [105]. In addition, the same authors reported that high consumption of vegetables reduced risk of BPH [105]. Similarly, data from HPFS study have demonstrated that consumption of fruits and vegetables rich in *β*-carotene lutein or vitamin C was inversely related to BPH [106]. As reported above, oxidative damage and inflammation are thought to be associated with development of BPH. High consumption of unsaturated fatty acids might contribute to lipid peroxidation of the cell membrane exacerbating the inflammation and impairing 5*α*-reductase activity [107]. Conversely, high intake of fruits and vegetables was found to be associated with less oxidative stress, as measured by malondialdehyde concentration [108]. The effect of diet on BPH/LUTS is also supported by the lower incidence of prostate related problems in some Asian countries using a predominantly plat-based diet, as compared with Western countries, using a provokingly animal-based diet [109].

Physical activities were also shown to reduce the possibility of prostate enlargement, LUTS, and LUTS-related surgery [110]. In particular, increasing walking by 3 h/week decreases the risk of BPH by 10% [110]. In a meta-analysis that enrolled 43,083 male patients, intensity of exercise was related to reduction of risk of prostate enlargement. Compared to the sedentary group, the risk for BPH or LUTS was significantly reduced with OR = 0.70, 0.74, and 0.74 for men engaging in light, moderate, and heavy physical activity, respectively [111].

In conclusion, type of diet and level of physical activity are emerging as other important factors affecting prostate health in the aging male, most probably reducing risk factors such as MetS, hypogonadism, and inflammation. However, whether physical exercise, weight loss, and modifications of dietary habit can really alter the natural history of BPH/LUTS remains to be determined. Further research is advisable to better clarify these points.

### 5.2. PDE5 Inhibitors and BPH/LUTS

**Figure 3:**
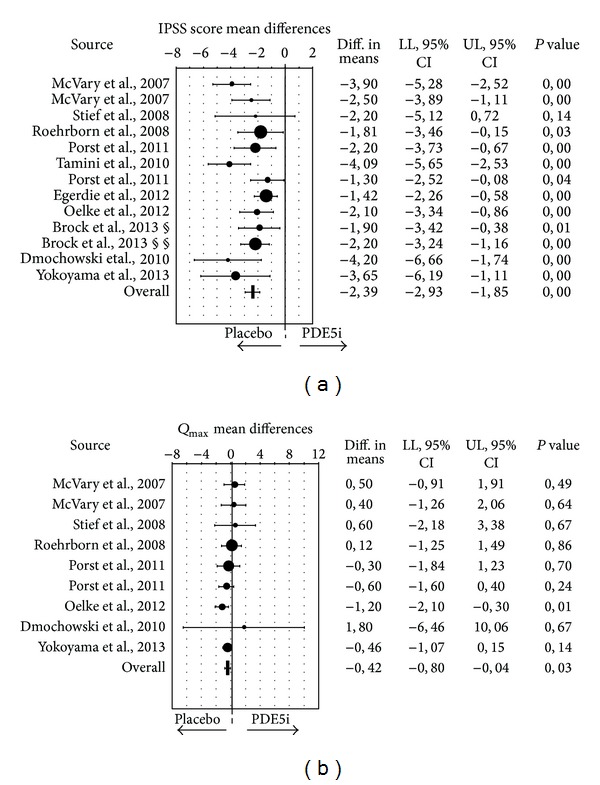
Weighted differences (with 95% confidence interval (CI)) of International Prostate Symptom Score (IPSS; (a)) and maximum flow rate (*Q*_max⁡_; (b)), for the studies on phosphodiesterase type 5 inhibitors (PDE5-Is) versus placebo. § no erectile dysfunction; §§ erectile dysfunction.

Emerging evidence suggests that PDE5i might reduce moderate-to-severe (storage and voiding) LUTS in men with or without ED [98]. Accordingly, tadalafil (5 mg once daily) has been approved by the US Food and Drug Administration (FDA) and by the European Medical Agency (EMA) and licensed for the treatment of male LUTS in Europe. By meta-analyzing the available evidence we previously reported that PDE5i alone, as compared with placebo, is able to improve LUTS symptoms, as detected by the decrease of IPSS score [112]. In addition, the association of PDE5i and *α*1-adrenergic blockers improved both IPSS score and maximum urinary flow rate (*Q*_max⁡_) at the end point, as compared with *α* blockers alone [112]. Since our last analysis, other five double-blind, placebo-controlled, randomized clinical trials (RCT) comparing the effect of PDE5i versus placebo on BPH/LUTS, have been published (see Table 2). Hence, so far, 12 RCTs [113–124] have specifically evaluated the effect of PDE5i alone in patients with BPH/LUTS. Overall, the studies enrolled 5158 patients, with a mean follow-up of 11.6 weeks (Table 2). Similar to previous analysis [112], we now report that PDE5i treatment was associated with a significant improvement of LUTS, as detected by the reduction of total IPSS score (Figure 3(a)). In addition, present meta-analysis also originally shows that PDE5i users report a small, but significant, improvement in *Q*_max⁡_ (Figure 3(b)). Hence, the current analysis, in a larger cohort of subjects, further indicates a role of PDE5i in improving LUTS in patients with BPH. In addition, it shows for the first time a possible role of PDE5i in improving urinary outflow in the same category of subjects.

Despite the aforementioned clinical evidence, the mechanism of action (MOA) of this class of medication in BPH/LUTS is still a matter of debate. Several dedicated recent reviews are available on this topic [112, 125–127]. Preclinical studies demonstrated that prostate, bladder, and urethra, as well as their relative blood vessels, all represent potential targets of PDE5i [128, 129]. Experimental evidence suggested that chronic blockade of PDE5 could impact several pathways involved in LUTS generation [88, 112, 125–127], including a reduced nitric oxide (NO)/cyclic guanosine monophosphate (cGMP) and an increased RhoA/Rho-kinase signalling [130–132]. In addition, PDE5i can also reduce chronic pelvic hypoxia and its related functional and morphologic changes in the bladder and prostate, by increasing blood perfusion [129, 133]. A possible direct effect for PDE5i in modulating autonomic nervous system overactivity and bladder/prostate afferent nerve activity was also suggested [134]. However, in the last few years, some experimental and clinical data have offered a new MOA for PDE5i in BPH/LUTS, reducing MetS-associated prostate inflammation. In the previously described rabbit model of MetS-associated prostate alterations we found that tadalafil dosing was able to reduce inflammation and leukocyte infiltration (CD 45 scoring), along with fibrosis/myofibroblast activation [97]. In a retrospective pilot study on a BPH population (*n* = 60), previously enrolled in a double-blind, placebo-controlled, clinical study on the efficacy of daily vardenafil (10 mg) added to tamsulosin (0.4 mg) [135], we evaluated prostatic CD 45 score in those undergoing simple prostatectomy for persistent/recurrent severe urinary symptoms. Patient cohort was categorized according to the presence of MetS. In those without MetS, CD45 positivity was low and unaffected by vardenafil dosing. In those with MetS, increased CD45 positivity was significantly blunted by chronic vardenafil treatment [88]. It is interesting to note that even in this small cohort the MetS factor most closely associated with CD45 positivity was dyslipidemia. Interestingly, in isolated human BPH stromal cells both tadalafil and vardenafil decreased TNF*α*-induced expression of genes related to inflammation (COX-2, IL-8, Il-6, IP-10, and MCP-1) and tissue remodelling (*α*SMA, bFGF). Similar results were obtained when TNF*α*-induced secretion of IL-8 and CXCR-10 was considered. Both vardenafil and tadalafil were able to blunt IL-8 secretion induced also by metabolic stimuli, such as oxLDL, AGE, and IGF-1. The effect was apparently due to the ability of these PDE5i to stimulate PKG activity because it was mimicked by a nonhydrolysable cGMP analog and blocked by a PKG antagonist. Finally, both PDE5i significantly reduced the ability of TNF*α* to increase the expression of oxLDL receptor, LOX-1 [88].

## 6. Conclusions

**Figure 4:**
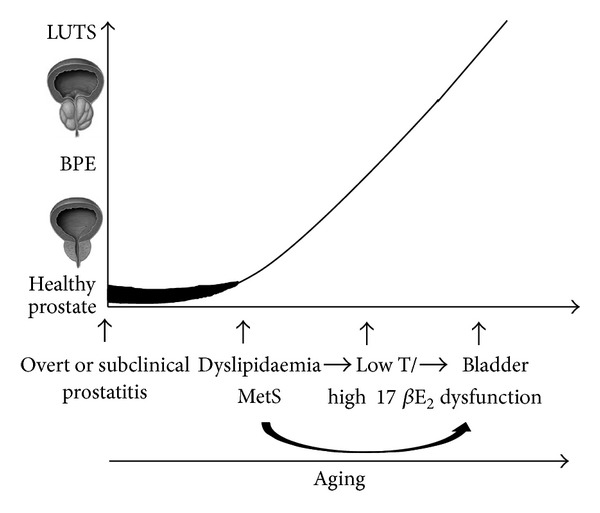
Graphical representation of a proposed multifactorial pathogenesis of benign prostatic hyperplasia/low urinary tract symptoms (BPH/LUTS). Symptomatic or asymptomatic prostate inflammation (very common in young individuals), in the presence of permissive factors such as metabolic syndrome (MetS), and in particular dyslipidemia, or an altered sex steroid milieu, can progress through prostate enlargement (BPE). The latter can or cannot be associated with LUTS, in particular in the presence of bladder dysfunction. Recent data indicates that MetS itself can also favour bladder alteration.

People are living longer and, in some parts of the world, healthier lives. In 2006, almost 500 million people worldwide were 65 and older. By 2030, that total is projected to increase to 1 billion—1 in every 8 of the earth's inhabitants. Significantly, the most rapid increases in the 65 and older population are occurring in developing countries, which will see a jump of 140 percent by 2030 [136]. Hence, we must proactively face the health issues of the elderly. BPH/LUTS represent significant bother among aging men; they were historically considered as a “normal” consequence of the aging process and, as such, their negative effects on men's well-being only dealt with through medical or surgical intervention. This view has been challenged in the last decade and now BPH/LUTS are seen more as preventable than inexorable ailments of the elderly [137]. Evidence presented in this review indicates that several modifiable metabolic factors play a role in the determinism or progression of LUTS/BPH. MetS and its components, hypogonadism, and prostate inflammation are, in fact, emerging as medical conditions commonly associated with BPH/LUTS.  Figure 4 summarizes our view. In our view, BPH/LUTS may be viewed as a complex disorder that also involves a metabolic component that may begin early in the life of the male, and, although asymptomatic, it is likely detectable even in the early stages of the disease. The mechanisms underpinning the relationship between MetS and prostate inflammation are likely to be similar in young and old men but chronic exposure to elevated inflammation, along with low T/high 17*β*E2, may contribute to BPH in the long term. Preventing the development of the disease even from the asymptomatic phase should be the basis for designing a resilient program of elderly healthcare. Analysis of the European Prospective Investigation into Cancer and Nutrition (EPIC)-Norfolk cohort has clearly demonstrated that the adverse CV effects of having MetS on coronary heart disease could be substantially reduced or nullified by increasing physical activity. Several epidemiological studies support this view also for BPH/LUTS; intervention studies are urgently needed.

## Figures and Tables

**Table 1:** Characteristics of the studies comparing International Prostatic Symptom score (IPSS) and prostate volume in patients with and without metabolic syndrome (MetS) according to different criteria. NCEP-ATPIII: National Cholesterol Education Program-Third Adult Treatment Panel; IDF: International Diabetes Federation; AHA/NHLBI: American Heart Association/National Heart, Lung, and Blood Institute.

Overall population			Men with MetS			Men without MetS		
Study	MetS criteria	Age (years)	Number of pts	IPSS total score	Prostate volume (cc)	Number of pts	IPSS total score	Prostate volume (cc)
Ozden et al., 2007 [67]	NCEP ATP III	60	38	22	37.4	40	20	32
Park et al., 2008 [68]	NCEP ATP III	74 ± 8.1	102	11.1	41.7	246	12.3	40.4
Yim et al., 2011 [69]	NCEP ATP III	41.4 ± 5.2	140	—	18.4	708	—	17.8
Jeong et al., 2011 [70]	NCEP ATP III	46.4 ± 8.4	354	6.8	20.6	1003	6.5	19.7
Byun et al., 2011 [71]	NCEP ATP III	55.6 ± 9.72	209	6.85	31.4	499	7.89	29.8
Yang et al., 2012 [72]	NCEP ATP III	53.8 ± 6.9	142	—	30.1	278	—	25.2
Gacci et al., 2013 [4]	AHA/NHLBI; IDF	68.2 ± 7.4	86	22.5	63	185	20.9	58
Park et al., 2013 [73]	NCEP ATP III	50–59	355	10	34	869	10	28

**Table 2:** Characteristics of the studies included in the meta-analysis.

Overall population						
Study	Age (years)	Duration (weeks)	Drugs	Dosage (mg)	Placebo number of pts	PDE5 number of pts
McVary et al., 2007 [113]	60	12	Sildenafil	50 (2 weeks); 100	155	168
McVary et al., 2007 [114]*	61.5	12	Tadalafil	5 (6 weeks); 20	126	125
Stief et al., 2008 [115]	55.9	8	Vardenafil	10	110	105
Roehrborn et al, 2008 [116]*	62.0	12	Tadalafil	2.5; 5; 10; 20	185	701
Porst et al., 2009 [117]*	61.9	12	Tadalafil	2.5; 5; 10; 20	105	386
Tamimi et al., 2010 [118]*	60.9	12	UK-369003	10; 25; 50, 100	37	246
Porst et al., 2011 [119]	64.8	12	Tadalafil	5	152	148
Egerdie et al., 2012 [120]*	62.5	12	Tadalafil	2.5; 5	200	406
Oelke et al., 2012 [121]	63.6	12	Tadalafil	5	172	171
Brock et al., 2013 [122]	63.3	12	Tadalafil	5	545	544
Dmochwski et al., 2013 [123]	58.6	12	Tadalafil	20	101	99
Yokoyama et al., 2013 [124]*	63.2	12	Tadalafil	2.5; 5	154	306

*The effect derived from a ponderated mean at end point on International Prostate Symptom Score and maximum urinary flow rate were analyzed.
